# Vitamin D deficiency associations with metabolic, bone turnover and adverse general health markers in community free living adults

**DOI:** 10.1186/s12902-021-00926-z

**Published:** 2022-01-06

**Authors:** Salah Gariballa, Javed Yasin, Ghada Abluwi, Awad Al Essa

**Affiliations:** Internal Medicine, Faculty of Medicine & Health Sciences, United Arab Emirates University, PO Box 17666, Al Ain, United Arab Emirates

**Keywords:** Vitamin D, Bone turnover, Inflammation, Metabolic risk factor

## Abstract

**Background:**

Although there is some evidence that vitamin D deficiency is highly prevalent in the Middle East, however its health impact is still not clear. The aim of this study was to assess the prevalence, causes and health implications of vitamin D deficiency in local United Arab Emirates (UAE) citizens.

**Methods:**

A cross-sectional study was conducted on community free living adults living in the city of Al Ain, UAE. Following informed written consent eligible subject’s blood and urine samples were taken for measurements of vitamin D [25(OH)D], metabolic and bone turnover markers. Clinical assessment that includes general and self-rated health, muscle health, and physical activity were also performed.

**Results:**

A total of 648 subjects (491 female) were included in this analysis. Their mean (SD) age was 38 (12) years. Mean 25(OH)D was 24 ng/ml (range: 4–67) with 286 (44%) subjects found to have vitamin D deficiency (< 20 ng/ml), 234 (36%) subjects have insufficiency (20-32 ng/ml) and 128 (20%) subjects have optimal concentrations (> 32 ng/ml). 25(OH)D concentrations were significantly higher in local indigenous UAE subjects compared to other Arab expatriates (*p* = 0.071). Although there were no statistically significant differences in clinical markers between groups, however, utra-sensitive C-reactive protein (us-CRP), parathyroid hormone (PTH), body mass index (BMI) and the bone markers U-PYD and PYD/CR were higher in vitamin D deficient older subjects aged ≥50 years and female subjects younger than 50 years respectively compared to those with insufficiency or optimal concentrations (*p* value < 0.05. Multiple logistic regression analysis revealed significant and independent association between 25(OH)D status and age and sex (*p* < 0.05).

**Conclusion:**

Older subjects with vitamin D deficiency have increased BMI, inflammation and PTH compared with those with insufficiency or optimal concentrations. Co-existence of obesity and vitamin D deficiency may have increased adverse health effects.

## Background

Vitamin D an active hormone is essential for both bone and muscle health [1]. There is also mounting evidence that vitamin D can convey other health benefits because of the discovery that most cells and tissues in the human body contain vitamin D receptors [1]. Indeed, many observational studies have linked vitamin D deficiency or insufficiency to increased risk of cardiovascular, malignant, autoimmune and infectious diseases [1]. There is also some evidence that vitamin D deficiency is associated with increased inflammation present in obesity and related chronic diseases [2]. Humans get vitamin D from exposure to sunlight, from diet, and from dietary supplements. Other factors such as age, skin pigmentation, smoking and adiposity are known to affect vitamin D levels [1, 3]. Serum 25-hydroxyvitamin D [25(OH)D] is considered the best indicator for vitamin D status and a predictor of bone health and also independent predictor of risk of a number of other chronic diseases [4]. A 25(OH)D concentration of 32 ng/mL (80 nmol/L) is proposed to be optimal for health based on variables of bone health [5]. Several studies have revealed that vitamin D deficiency is common in the Middle East and the Indian subcontinent and appears largely to be due to inadequate sun exposure [6–8]. However most of these studies so far were of small size. Furthermore, other causes and more importantly the health Implications of low vitamin D status are not clearly defined. Indeed, many studies on the relationship between low vitamin D status and bone health in south Asian population have yielded conflicting results [7, 8]. Furthermore, some cross-sectional studies comparing Caucasian and South Asian postmenopausal women point to altered metabolism of vitamin D in the Asian women that may protect their skeleton from bone loss. Alternatively, ethnic and/or genetic difference may influence the capacity to synthesize vitamin D [5, 9]. Although some studies have reported a high prevalence of vitamin D deficiency among UAE citizens, however the clinical benefit of optimizing vitamin D levels is still lacking. The aim of this study was to assess the prevalence, determinants and whether there is measurable increase in markers of bone turnover and adverse health outcomes in 25(OH)D deficient UAE subjects.

## Methods

Details of the study participants and methods were published before [10]. Briefly participants in this study includes Emirati (UAE citizens) and expatriates from other Arab countries aged 18 years and over. They were part of a trial to assess the clinical benefit of vitamin D3 supplements. Participants were recruited from community health centers and local hospitals. The exclusion criteria include those taking calcium and/or vitamin D supplementation, bisphosphonates, steroid medications, hormones or diuretics. Subjects diagnosed with renal disease or stones, hypercalcaemia, and those unable to give an informed written consent were also excluded. Blood and urine samples were taken from eligible subject’s for measurements of 25(OH)D, biochemical metabolic and bone turnover markers and related biochemical variables following an informed written consent. Information on self-rated health, muscle pains, physical activity and dietary intakes were also collected at baseline.

### Demographic, clinical and anthropometric measurements

Lifestyle and health factors relevant to vitamin D deficiency were collected using a face-to-face questionnaire. Collected data included information on education and socio-economic status, current and past occupation, tobacco smoking, history of previous illness, sun exposure, body pains, use of herbal medicine, vitamin supplements, exogenous hormones for contraception and postmenopausal replacement therapy. Dietary information was obtained by a food frequency questionnaire [11]. A validated questionnaire was used to assess occupation and leisure-related physical activity [12]. Height, weight and blood pressure were measured using standard methods. Fasting blood & urine samples were also collected. Average time of sun exposure during the day and body parts directly exposed to the sun was assessed using a face-to-face questionnaire.

### Biochemical and urine analysis

Biochemical analyses included 25(OH)D, total P1NP (total Procollagen type 1 amino-terminal propeptide), Osteocalcin (OCN) and PTH (Parathyroid Hormone) = were measured using fully automated COBAS e411 analyzer that uses a patented Electro Chemi Luminescence (ECL) technology for immunoassay analysis) from ROCHE diagnostics, Manheim; U PYD = Human Pyridinoline (PYD) measured using ELISA Kit from MyBioSource, USA Cat. No. MBS030461: U DPD = Human Deoxypyridinoline (DPD) measured by ELISA Kit from MyBioSource, USA Cat. No. MBS039364; CTX-1 = Human cross-linked carboxy-terminal telopeptide of type I collagen measured using ELISA Kit Cat. No. MBS700254. Routine baseline tests including Cholesterol, Triglyceride, HbA1c, blood Glucose, hs-CRP, calcium and phosphorus, urinary creatinine were measured using Integra 400 plus Auto Analyzer from ROCHE diagnostics, Manheim.

### Statistical analysis

SPSS software, version 25.0 (SPSS Inc., Chicago) was used. 25(OH)D concentrations were divided into 4 equal quartiles & deficiency/insufficiency/optimal levels based on biochemical normative values accepted by many International societies [vitamin D deficiency (< 20 ng/ml), insufficiency (20-32 ng/ml) and optimal concentrations (> 32 ng/ml)] [5]. Analysis of variance (ANOVA) or the nonparametric Kruskal-Wallis H test was used to test for within and between-group differences. The influence of prognostic indicators including age, sex, body mass index, calcium & vitamin D dietary intakes, sun exposure, smoking and physical activity on vitamin D status (deficiency vs. insufficiency vs optimal) was examined using multiple regression analysis.

## Results

Six hundred forty-eight subjects (491 female) were included in this analysis. Their mean (SD) age was 38 (12) years. Mean 25(OH)D concentration was 24 ng/ml (range: 4–67) with 286 (44%) subjects found to have vitamin D deficiency (< 20 ng/ml), 234 (36%) subjects have insufficiency (20-32 ng/ml) and 128 (20%) subjects have optimal concentrations (> 32 ng/ml).

**Fig. 1:**
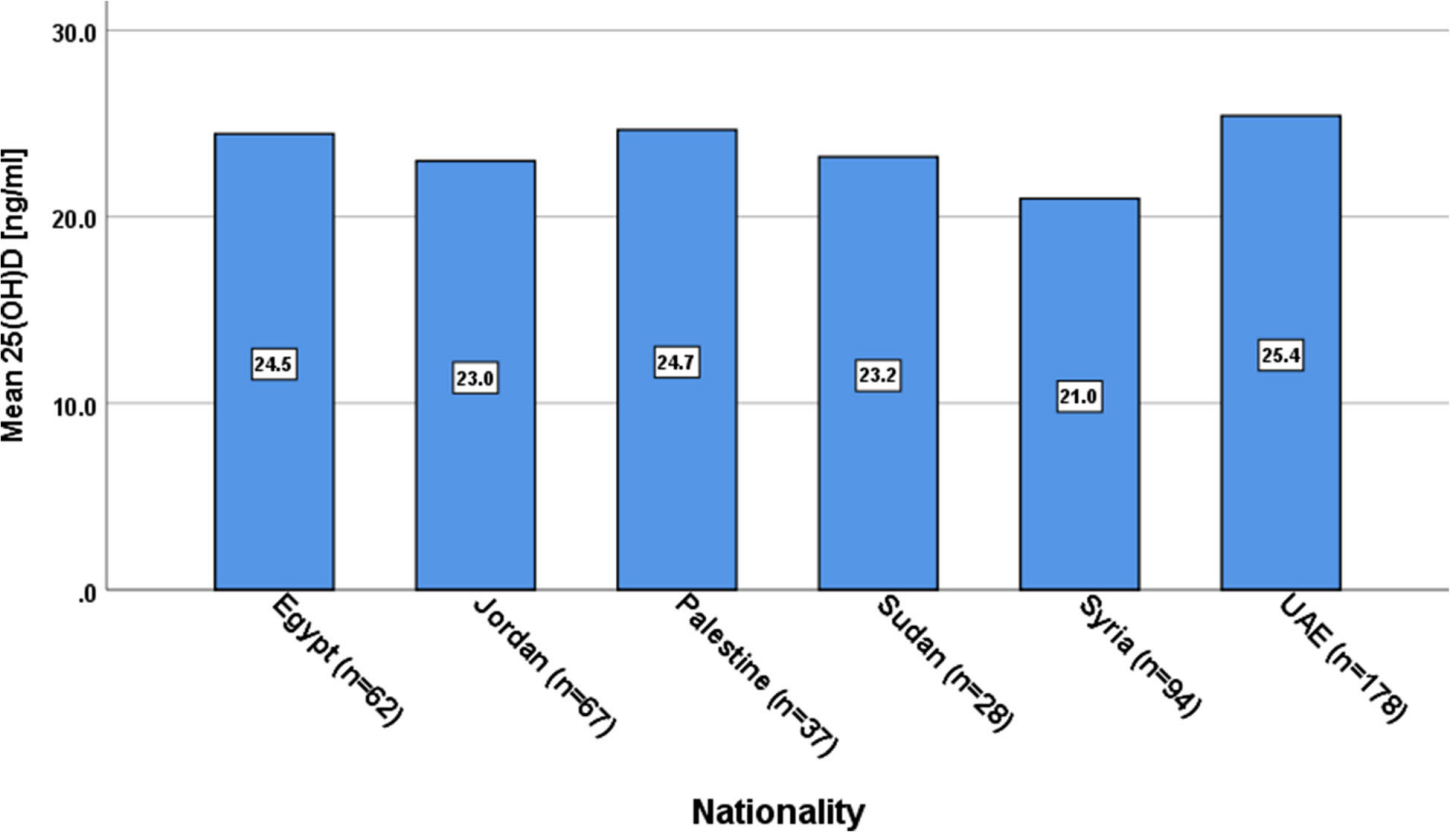
Baseline 25(OH)D concentrations according to study population Nationally. *P* value = 0.071 for between groups difference

Figure 1 shows baseline Vitamin D levels for local UAE subjects and expatriates from other Arab countries. We found statistically significant differences in 25(OH)D concentrations between ethnic Arab expatriates and local indigenous UAE subjects (*p* = 0.071).

We also found significant differences in age, sex and prevalence of diabetes mellitus between subjects with 25(OH)D deficiency compared to those with higher concentrations with higher number of females and younger subjects in those with lower concentrations (Table 1).

**Table 1:** Baseline characteristics of subjects according to serum 25(OH)D concentrations [mean (SD)], unless stated otherwise

Characteristic	Deficiency 25(OH)D (< 20 ng/ml)	Insufficiency 25(OH) (20-32 ng/ml)	Optimal 25(OH)D (> 32 ng/ml)	*P value*
Total number of subjects n (%)	286 (44)	234 (36)	128 (20)	
Age (years)*	36.5 (12)	38.7 (12)	41.4 (13.0)	0.002
Age < 50, n (%)	239 (49)	172 (35)	77 (16)	
Age ≥ 50, n (%)*	39 (32.5)	45 (37.5)	36 (30)	0.000
Sex				
Female*	233 (82)	168 (72)	88 (69)	0.004
Do you smoke, n (%)				
Yes, on most or all days	33 (11.8)	31 (13.4)	16 (12.9)	
Only occasionally	10 (3.6)	10 (4.3)	8 (6.5)	
No	236 (84.6)	190 (82.3)	100 (80.6)	0.723
Diabetes mellitus*				
Yes	29 (11.4)	26 (13.3)	23 (23.2)	0.015
Hypertension				
Yes	30 (11.8)	30 (15.3)	17 (16.7)	0.380
Angina or heart attack				
Yes	0 (0)	1 (0.5)	0 (0)	0.412
High cholesterol				
Yes	18 (7.1)	25 (12.8)	13 (13.3)	0.082
Sleep disturbance				
Yes	7 (2.8)	7 (3.6)	8 (8.3)	0.058
Past Operation				
Yes	133 (48.4)	88 (38.8)	44 (36.1)	0.017

*P value < 0.05 for between group difference

**Fig. 2:**
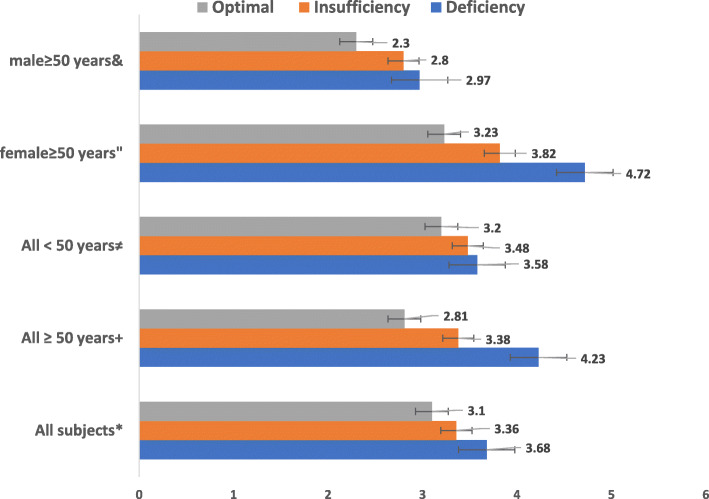
Baseline us-CRP (mg/L) according to serum 25(OH)D concentrations stratified by age and sex. & *p* = 0.682, “*p* = 0.251, ≠*p* = 0.745, + *p* = 0.061, * *p* = 0.002

**Fig. 3:**
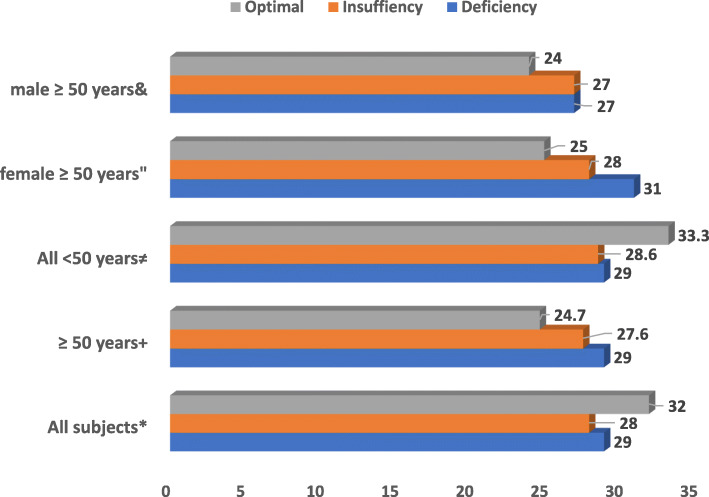
Baseline PTH (pg/ml) according to serum 25(OH)D concentrations stratified by age and sex. & *p* = 0.748, “*p* = 0.278, ≠*p* = 0.459, + *p* = 0.156, * *p* = 0.441

**Fig. 4:**
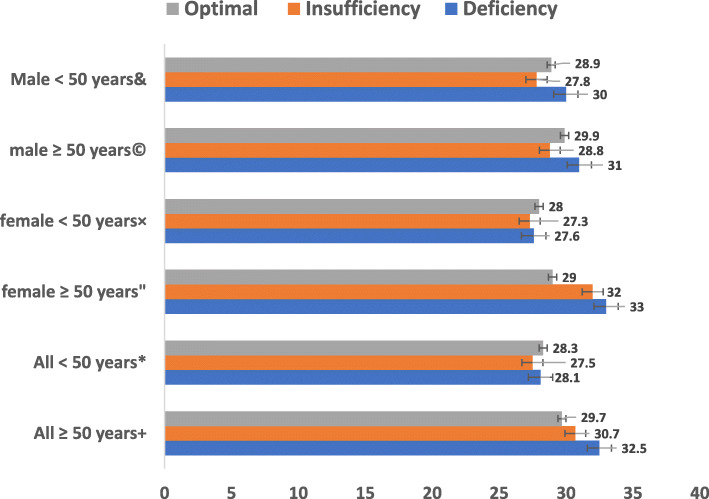
Baseline BMI according to serum 25(OH)D concentrations stratified by age and sex. & *p* = 0.093,© *p* = 0.348, ×*p* = 0.746, “*p* = 0.112, * *p* = 0.451, +*p* = 0.0.**0**41

Subjects with vitamin D deficiency (< 20 ng/ml) have increased us-CRP and PTH compared with those with insufficiency (20-32 ng/ml) or optimal concentrations (> 32 ng/ml) (Table 2, Figs. 2, 3). Us-CRP & PTH were higher in vitamin D deficient subjects particularly older ones compared with those with insufficiency or optimal concentrations (Figs. 2, 3). Figure 4 shows increased BMI for older subjects including females with vitamin D deficiency compared to those with insufficiency or optimal concentrations. 25(OH)D levels were also divided into 4 quartiles and analysed against a number of clinical and biochemical variables including inflammatory bone turn over measures (Table 3). No statistically significant differences were found between the two groups except in C-terminal telopeptide. Also, U-PYD and PYD/CR were higher in young female subjects with vitamin D deficiency compared with those with insufficiency or optimal concentrations (*p* value < 0.05). Multiple logistic regression analysis revealed significant and independent association between vitamin D status and age and sex only (*p* < 0.05), (Table 4). We found no statistically significant differences in clinical parameters including dietary intakes, physical activity, sun exposure or body pains and general health between subjects with highest vitamin D compared to those with the lowest concentrations (Tables 5 and 6).

**Table 2:** Clinical, anthropometric, biochemical markers of bone turnover and related variables of subjects according to serum 25(OH)D concentrations [mean (SD)] unless stated otherwise

Characteristic	Deficiency 25(OH)D (< 20 ng/ml)	Insufficiency 25(OH)D (20-32 ng/ml)	Optimal 25(OH)D (> 32 ng/ml)	*P value*
Weight (Kg)	77.8 (18)	78.76 (18)	80.81 (18)	0.313
BMI	29.47 (11)	29.13 (14)	28.89 (5)	0.910
SBP (mmHg)	122.8 (17)	125.7 (16)	124.2 (14)	0.320
DBP (mmHg) *	74.84 (8)	77.5 (8)	76.94 (8)	0.012*
HbA1C (%)	5.77 (0.97)	5.63 (0.90)	5.79 (1.13)	0.175
Glucose (mmol/L)	6.25 (2.67)	6.03 (2.44)	6.22 (2.73)	0.614
Hs CRP (mg/L)	3.68 (4.34)	3.36 (3.42)	3.10 (2.97)	0.326
Cholesterol (mmol/L)	4.82 (0.99)	4.88 (0.93)	4.78 (0.95)	0.598
HDL (mmol/L)	1.28 (0.73)	1.26 (0.39)	1.26 (0.36)	0.911
LDL (mmol/L)	3.26 (0.92)	3.26 (0.86)	3.11 (0.87)	0.246
Triglycerides (mmol/L)	1.34 (0.90)	1.45 (1.07)	1.39 (1.19)	0.457
Calcium (mmol/L)	2.29 (0.11)	2.28 (0.99)	2.29 (0.11)	0.679
Phosphate (mmol/L)	1.18 (0.20)	1.21 (0.17)	1.18 (0.19)	0.084
Osteocalcin (ng/ml)	18.0 (7.07)	18.11 (8.43)	17.97 (12.1)	0.986
PTH (pg/ml)	29.52 (18.5	28.72 (15.35)	32.37 (48.2)	0.441
P1NP (ng/ml)	50.22 (23)	47.71 (21.48)	50.85 (59.8)	0.600
CTX1 (ng/ml)	10.17 (7.3)	11.83 (7.58)	10.41 (6.56)	0.212
U-DPD (nmol/L)	95.57 (47.8)	90.81 (44.9)	93.89 (48.6)	0.837
U-PYD (nmol/L)	230.14 (89.6)	234.11 (104)	217.38 (93)	0.670
PYD/Cr (nmol/mmol)	24.14 (11.6)	24.34 (12)	22.61 (11.8)	0.738
DPD/Cr (nmol/mmol)	10.29 (6.03)	9.63 (5.66)	10.13 (6.5)	0.806

*P < 0.05

*Abbreviations*: *HDL* High density lipoprotein, *LDL* Low density lipoprotein, *HsCRP* High sensitivity C reactive protein, *PTH* Parathyroid Hormone, *P1NP* Procollagen type-1 N-terminal propeptide, *CTX1* C-terminal telopeptide of type −1 collagen, *U DPD* Urine Deoxypyridinoline, *U PYD* Urine Pyridinoloine, *OSTEO* Osteocalcin

**Table 3:** Anthropometric, biochemical markers of bone turnover and related variables of subjects according to serum 25(OH)D concentrations divided into 4 equal quartiles [mean (SD)]

Variable	1st quartile 25(OH)D ≤15 ng/ml	2nd quartile 25(OH)D =15.1–21.9 ng/ml	3nd quartile 25(OH)D =22–30 ng/ml	4th quartile 25(OH)D > 31.1 ng/ml	*P* value
Weight (Kg)	77.26 (17)	79.51 (18)	78.50 (17)	79.67 (17)	0.622
BMI	28.93 (5)	28.98 (5)	28.07 (4)	28.31 (5)	0.423
SBP (mmHg)	121.83 (16)	125.62 (18)	124.27 (15)	124.67 (13)	0.397
DBP (mmHg)	74.67 (8)	75.78 (7)	77.34 (8)	77.06 (7)	0.089
HbA1C (%)	5.72 (0.9)	5.84 (1)	5.60 (0.7)	5.72 (1)	0.196
Glucose (mmol/L)	6.14 (2)	6.48 (3)	6.02 (1)	6.00 (2)	0.321
Hs CRP (mg/L)	3.97 (4)	3.38 (3)	3.33 (3)	3.10 (2)	0.198
Cholesterol (mmol/L)	4.79 (0.9)	4.88 (0.9)	4.88 (0.9)	4.75 (0.9)	0.541
HDL (mmol/L)	1.32 (0.9)	1.23 (0.4)	1.27 (0.3)	1.25 (0.3)	0.494
LDL (mmol/L)	3.22 (0.9)	3.31 (0.8)	3.26 (0.8)	3.11 (0.8)	0.239
Triglycerides (mmol/L)	1.27 (0.8)	1.48 (1)	1.44 (1)	1.36 (1)	0.249
Calcium (mmol/L)	2.28 (0.1)	2.29 (0.1)	2.28 (0.1)	2.28 (0.1)	0.881
Phosphate (mmol/L)	1.17 (0.2)	1.19 (0.1)	1.21 (0.1)	1.18 (0.1)	0.369
Osteocalcin (ng/ml)	17.92 (6)	18.10 (7)	17.45 (7)	18.66 (11)	0.666
PTH (pg/ml)	28.70 (18)	30.62 (18)	27.73 (15)	32.10 (43)	0.446
P1NP (ng/ml)	50.10 (23)	49.90 (22)	46.75 (21)	50.98 (53)	0.682
CTX1 (ng/ml) *	11.58 (8)	9.12 (5)	12.45 (7)	10.41 (6)	0.034*
U-DPD (nmol/L)	101.53 (45)	89.08 (47)	88.53 (43)	97.39 (50)	0.526
U-PYD (nmol/L)	232.94 (88)	234.62 (89)	221.59 (103)	227.71 (100)	0.914
PYD/Cr (nmol/mmol)	24.43 (10)	24.55 (12)	22.94 (11)	23.76 (12)	0.910
DPD/Cr (nmol/mmol)	11.05 (5)	9.39 (5)	9.44 (5)	10.50 (6)	0.534

*P < 0.05

**Table 4:** Multiple logistic regression analysis of the influence of some clinical prognostic variables on vitamin D status (deficiency vs. insufficiency/optimal) of study population

Variable	Odd ratio (95% C.I)	*P* value
Age (years)*	1.023 (1.004–1.041)	.015
Sex (male/female)*	.478 (.297–.770)	.002
BMI	.964 (.926–1.003)	.073
Sun exposure	1.012 (.929–1.102)	.791
Diet	1.023 (.951–1.101)	.545
Physical activity	1.065 (.787–1.441)	.684
Smoking	1.034 (.783–1.367)	.812
Diabetes mellitus	1.143 (.634–2.061)	.656
Hs CRP (mg/L)	.996 (.946–1.048)	.876
PTH (pg/ml)	1.001 (.994–1.008)	.718

*P < 0.05

**Table 5:** Self-rated health, body pains and sun exposure of subjects according to serum 25(OH)D concentrations [n(%)]

Characteristic	Deficiency 25(OH)D (< 20 ng/ml)	Insufficiency 25(OH)D (20-32 ng/ml)	Optimal 25(OH)D (> 32 ng/ml)	*P value*
Body pain n (%)				
No	182 (64.3)	138 (59.7)	80 (63)	
Yes	101 (35.7)	93 (40.3)	47 (37)	0,562
General health n (%)				
Excellent	42 (14.9)	32 (13.9)	16 (12.6)	
Good	178 (63.3)	140 (60.6)	76 (59.8)	
Fair	33 (11.7)	26 (11.3)	13 (10.2)	
Poor	16 (5.7)	23 (10.0)	15 (11.8)	
Do not know	12 (4.3)	10 (4.3)	7 (5.5)	0.231
Sun exposure: usual clothing when outdoors in the sun between 10:00 AM and 2:00 PM during each season during the past 12 months, n(%)				
Shorts & brief top with shoulders exposed	1 (0.4)	1 (0)	1 (1.0)	
Short and T-shirt or similar top	1 (0.4)	1 (0)	1 (1.0)	
Shorts and long sleeve	3 (1.2)	2 (0)	0 (0)	
Long pant and T-shirt or similar top	176 (69.8)	117 (0)	51 (53.1)	
Long pants and long sleeves	4 (1.6)	1 (0)	2 (2.1)	0.319
Sunscreen: describe your usual use of sunscreen when outdoors				
I almost never used sunscreen	5 (2.0)	4 (0)	5 (5.2)	
I used it occasionally	1 (0.4)	0 (0)	1 (1.0)	
I used it somewhat regularly	7 (2.8)	20 (0)	13 (13.5)	
I used it most of the time	0 (0)	1 (0)	0 (0)	
I used it all the time	1 (0.4)	1 (0)	0 (0)	0.856

*P value < 0.05

**Table 6:** Physical activity of subjects according to serum 25(OH)D concentrations [n (%)]

Characteristic	Deficiency 25(OH)D (< 20 ng/ml)	Insufficiency 25(OH)D (20-32 ng/ml)	Optimal 25(OH)D (> 32 ng/ml)	*P value*
Occupation: physical activity? n (%)				
Not very active	33 (11.8)	39 (17.0)	17 (13.8)	
Moderately active	157 (56.1)	120 (52.2)	62 (50.4)	
Very active	87 (31.1)	67 (29.1)	41 (33.3)	
Not working	3 (1.1)	4 (1.7)	3 (2.4)	0.464
Leisure: physical activity? n (%)				
Not very active	54 (19.1)	50 (21.7)	33 (26.2)	
Moderately active	193 (68.4)	140 (60.9)	76 (60.3)	
Very active	35 (12.4)	40 (17.4)	17 (13.5)	0.447
Physical activity till sweating n (%)				
Less than once per week	108 (38.8)	102 (44.5)	58 (46.4)	
1–2 times per week	54 (19.4)	43 (18.8)	20 (16.0)	
3–4 times per week	47 (16.9)	34 (14.8)	22 (17.6)	
5–6 times per week	43 (15.5)	29 (12.7)	15 (12.0)	
7–8 times per week	23 (8.3)	20 (8.7)	9 (7.2)	
More than 8 times per week	3 (1.1)	1 (0.4)	1 (0.8)	0.354

*P value < 0.05

## Discussion

Our results agree with previous studies from similar populations demonstrating a high prevalence of vitamin D deficiency. In addition, we found increased trends of increased BMI, inflammation and PTH in older subjects with biochemical deficiency or lowest vitamin D concentrations compared with those with biochemically normal or highest concentrations. We found no statistically significant association in biochemical bone turnover markers except for U-PYD and PYD/CR which are higher in young female subjects with vitamin D deficiency compared with those with insufficiency or optimal concentrations. In contrast no significant differences found in clinical parameters including dietary intakes, physical activity, sun exposure or body pains and general health between subjects with highest vitamin D versus those with the lowest concentrations. Vitamin D concentrations were higher in local indigenous UAE subjects compared to ethnic Arab expatriates. The association between low 25(OH)D levels and obesity (high BMI) and increased inflammation are interesting and may have public health importance particularly for our population. For example, abdominal obesity and related diabetes are very common in the UAE with the UAE having one of the highest prevalence in the World [13–16]. We have previously reported that abdominal obesity in our population is associated with increased inflammation and decreased antioxidant status [17]. The potential role of low plasma 25(OH)D concentrations as a mediator between obesity and increased risk of diabetes in the development of type 2 diabetes has been reported by several studies [18]. Experimental studies suggest 25(OH)D deficiency impairs glucose-induced insulin secretion and that insulin sensitivity may improve with 25(OH)D supplementation in patients with vitamin D deficiency [18–20]. Although, hitherto studies of the effects of vitamin D supplementations on obesity associated inflammation and risk of type 2 diabetes have produced conflicting results [21–23]. A recent study has reported that vitamin D supplementation to reach and sustain 25(OH)D levels of 100–124 and ≥ 125 nmol/l has been shown to lower the risk of progression to diabetes [24]. The results of this study encourage us to study the effects of higher dose, frequency and duration of vitamin D supplementation to reach and sustained higher levels to find out if indeed this will lead to a clinical benefit in our high-risk population. Currently ongoing trial results should provide us with more definitive answers.

The cutaneous manufacturing of 25(OH)D depends on a number of factors including age, skin color/pigmentation, season, latitude and exposure to sunlight including use of sunscreen. Furthermore, darker skin people require higher doses of sun exposure/ UV-B light to reach their maximum capacity of 25OHD production in the skin. Studies have reported that veiled Arabian women have lower 25OHD concentrations than non-veiled women [7]. We found no statistically significant differences in 25(OH)D concentrations between ethnic Arab expatriates and local indigenous Emirati subjects despite apparent differences in dress and behavioral life style practices including diet and physical activity between the two groups. A previous study of 259 women from the same population found no significant differences in 25(OH)D concentration between women who covered their whole body and those exposed their face and hands. However, the same study showed significantly lower 25(OH)D concentrations between local Emirati women and 7 non-Arab Caucasian ladies who dressed in a Western style [7]. Recent evidence suggest that sunlight exposure plays a much smaller role in maintaining vitamin D status in Asian population because of pigmented skin and/or altered metabolism and may therefore need increased oral intake of calcium and vitamin D [8]. In contrast to a previous study from the same population, we found no statistically significant association between vitamin D concentrations and dietary intakes [7]. A 1-year longitudinal study of 35 South Asian and 105 Caucasian women reported dietary intake of vitamin D had no impact on 25OHD levels [25]. Differences may be due to different dietary assessment tools used in different studies and/or inherent recall biases in dietary intakes assessment.

Vitamin D is also essential for both bone and muscle health. Although 2 previous studies in Arab men and women reported associations between 25(OH)D deficiency and increased muscle and back pains in the Saudi women and back aches disappeared with normalization of 25(OH)D levels, we did not however find statistically significant association in body pains and general health between subjects with highest 25(OH)D versus those with the lowest concentrations. A meta-analysis of studies on effects of vitamin D on muscle strength, mass and power reported a weak, but positive effects with 25(OH)D supplementations [26]. The effect was only evident in people aged 65 years or older [25]. However, a recent randomized controlled trial of 4 months vitamin D supplementation of 417 vitamin D deficient men and women aged 40–80 years from Norway did not show improvement in muscle strength [27]**.** The authors suggested age difference between the two study populations might be the reason for the discrepancy between the meta-analysis findings and their study. This may also be the reason for the lack of an association in our study in body pains and general health between subjects with highest 25(OH)D compared to those with the lowest concentrations. Given the known link between 25 (OH)D and muscle, health further research is needed to explore the relationship between vitamin D metabolism and musculoskeletal health.

Out of many bone turnover markers we measured in both blood and urine, only U-PYD and PYD/CR were significantly higher in young female subjects with 25(OH)D deficiency compared with those with insufficiency or optimal concentrations (*p* value < 0.05). Although we found a trend of increased PTH in both older male and females subjects with 25(OH)D deficiency (< 20 ng/ml) compared with those with insufficiency (20-32 ng/ml) or optimal concentrations (> 32 ng/ml), but these differences did not reach statistical significance. However, this does point to a degree of secondary hyperparathyroidism as a result of increased bone turnover associated with 25(OH)D deficiency. High PTH increase bone turnover and may exert inhibitory effects on bone growth by increasing the production of IGFBP and reducing IGF-1 [7]. Unfortunately, we did not measure either IGFBP or IGF-1. There is also some evidence that the relationship between PTH and 25(OH)D is not linear [28]. More importantly however, the threshold of 25(OH)D level in relation to bone turnover and other health outcomes for our citizens and much other similar Asian population is not definitely known. Research is clearly required to examine this important latter question.

Although we found higher concentrations of us-CRP in some vitamin D deficient subjects, however, the relationship between 25(OH)D and inflammatory markers has not been consistent [29]. A meta-analysis of trials that examined the impact of vitamin D supplementation on inflammatory biomarkers have also reported conflicting results [30, 31]*.* This was because some of the trials included in the meta-analysis were of small sample size with participants of wider age groups and comorbidities [29]. Larger studies with longer follow up may help to clarify the relationship between vitamin D levels and inflammatory pathways.

## Conclusion

Our results point to an association between 25OHD deficiency and increased metabolic risk factors. Current evidence from other population also show a plausible link between low 25OHD and obesity and associated inflammation and type 2 diabetes, but whether this association is causal remains unclear. Ongoing trial results should provide us with more answers to these important questions. However, given the co-existence of pathologically high prevalence of 25OHD deficiency and obesity in our population the potential benefits of optimizing vitamin D status is likely to have significant health implications. Hence the need for this research in ours and other similar high-risk populations.

## Data Availability

Data is available upon request to the corresponding author.

## Abbreviations

HDL: High density lipoprotein
LDL: Low density lipoprotein
HsCRP: High sensitivity C reactive protein
PTH: Parathyroid Hormone
P1NP: Procollagen type-1 N-terminal propeptide
CTX1: C-terminal telopeptide of type − 1 collagen
U DPD: Urine Deoxypyridinoline
U PYD: Urine Pyridinoloine
OSTEO: Osteocalcin
